# Antibiotics in early life and childhood pre-B-ALL. Reasons to analyze a possible new piece in the puzzle

**DOI:** 10.1007/s12672-022-00465-6

**Published:** 2022-01-10

**Authors:** T. M. Cardesa-Salzmann, A. Simon, N. Graf

**Affiliations:** Department of Pediatric Hematology and Oncology, Universitätsklinikum des Saarlandes, Homburg, Saarland Germany

**Keywords:** Childhood acute lymphoblastic leukemia, Inherited susceptibility, Microbiome, Antibiotics, Innate immune response, Inflammation, Leukemia microenvironment

## Abstract

Acute lymphoblastic leukemia (ALL) is the most common pediatric cancer with precursor B-cell ALL (pB-ALL) accounting for ~ 85% of the cases. Childhood pB-ALL development is influenced by genetic susceptibility and host immune responses. The role of the intestinal microbiome in leukemogenesis is gaining increasing attention since Vicente-Dueñas’ seminal work demonstrated that the gut microbiome is distinct in mice genetically predisposed to ALL and that the alteration of this microbiome by antibiotics is able to trigger pB-ALL in *Pax5* heterozygous mice in the absence of infectious stimuli. In this review we provide an overview on novel insights on the role of the microbiome in normal and preleukemic hematopoiesis, inflammation, the effect of dysbiosis on hematopoietic stem cells and the emerging importance of the innate immune responses in the conversion from preleukemic to leukemic state in childhood ALL. Since antibiotics, which represent one of the most widely used medical interventions, alter the gut microbial composition and can cause a state of dysbiosis, this raises exciting epidemiological questions regarding the implications for antibiotic use in early life, especially in infants with a a preleukemic “first hit”. Sheading light through a rigorous study on this piece of the puzzle may have broad implications for clinical practice.

## Genetic susceptibility and exogenous exposures

Childhood pB-ALL development is influenced by a combination of genetic susceptibility and exogenous factors.

Germline predisposition is an important risk factor underlying the development of pediatric ALL [1, 2], present in 3–10% of childhood ALL cases [3]. In pB-ALL the genetic susceptibility ranges from frequent but low-penetrant prenatal somatic chromosomal aberrations [4–8] and adverse combinations of low penetrant germline variations [2, 9], to rare but highly penetrant germline mutations [10].

Germline variations predisposing to ALL include constitutional syndromes like Down syndrome[11], Noonan syndrome[12], familial cancer syndromes, Li-Fraumeni syndrome[13], constitutional mismatch repair deficiency syndrome[14, 15]), *PAX5* mutations[16, 17], *ETV6* variants [18, 19] as well as genes harboring germline non-silent variants presumed to confer a risk of sporadic ALL like *NBN*, *FLT3*, *SH2B3*, and *CREBBP* [2, 10].

The most frequent fusion gene in pediatric ALL, resulting from t(12;21)(p13.2;q22.1), is the *ETV6-RUNX1* fusion, which occurs in approximately 25% of childhood pB-ALL [3]. Pre-leukemic clones carrying *ETV6-RUNX1* oncogenic lesions have been found in 1–5% of healthy newborn cord bloods [20, 21], with differences based mainly on different detection methods, with at an estimated frequency that was 100–500-fold greater than the risk of the corresponding leukemia [7, 20–24]. Prenatal low penetrant somatic chromosomal alterations in pB-ALL include the *ETV6-RUNX1* fusion [4], high hyperdiploidy [5], the *TCF3-PBX1* gene fusion [6, 25] and *BCR-ABL1* fusions [8]. Why only a fraction of children born with these preleukemic “hits” develop full-blown leukemia is still unknown, highlighting the fact that a second oncogenic step is required for overt leukemia development.

Although childhood pB-ALL does not generally cluster geographically, various pathogens have been reported linked to pB-ALL in space–time clusters, such as peaks after seasonal influenza [26], adenoviral infection [27], and H1N1 infection clusters [28]. Greaves hypothesized that while microbial exposures earlier in life are protective, in their absence delayed exposure of an immature untrained immune system to infection results in altered immune responses triggering critical secondary mutations [20]. Epidemiological factors linked to a protective effect for ALL in children less than one year old include birth order [29], mode of delivery [30–33], breastfeeding [34], early day care attendance [35–39], early common infections [40] and animal contact [41], supporting the hypothesis that infections have a protective effect for ALL in children less than one year of age [42]. Concomitant with rising hygiene standards, the incidence of pB-ALL has increased in developed countries [43–46]. More hygienic environments may reduce the chance to properly train the immune system to prevent secondary mutations during later infections. Modulation of the innate and adaptive immune system by vaccines has been associated to protection from ALL, in particular early BCG vaccination before 3 months of age [47]. The Bacillus Calmette-Guérin (BCG) vaccine is a live attenuated tuberculosis vaccine that has the ability to induce non-specific cross-protection against pathogens that might be unrelated to the target disease. A meta-analysis of 12 studies observed that early vaccination (< 3 months of age) with the Bacillus Calmette–Guérin (BCG) vaccine results in statistically robust protection from ALL [47]. In line with this observation, differences in vaccination protocols of BCG vaccination in East and West Germany prior to reunification, with compulsory BCG vaccination in East but not in West Germany, correlated with a lower rate of childhood leukemia in East Germany before reunification, which increased to West German levels 8 years after reunification [48].

## Insights from preclinical pB-ALL models

The “innate immune memory” or “trained immunity” immunologic principle highlights the responses of innate immune cells and their memory properties after immune stimuli [49]. Macrophages, monocytes and NK cells undergo metabolic and epigenetic modifications following exposure to infection or vaccination, providing innate immune cells with a memory, which subsequently modulates their response to a second infection exposure later in life [50]. The lasting immunologic memory is mediated by persistent epigenetic modifications in hematopoietic stem cells (HSC) and myeloid progenitors and depends on the transcription factor CCAAT/enhancer-binding protein b [51]. When cells of the hematopoietic niche are trained with beta-glucan and BCG, they generate accumulation of H3K4me3 and H3K27ac histone complexes, which are partially preserved when HSC differentiate along myeloid and lymphoid lineages [52]. A new mechanism of inherited trained immunity has been described in mice [53], conferring further protection against infections to the offsping. Innate host immune responses can influence the penetrance of B-ALL. The protective role of early immune training in B-ALL progression even in the presence of first hits was explored by Fidanza et al. in Eμ-ret and *E2A-PBX1* transgenic murine B-ALL models [54]. Ex vivo stimulation of leukemia-initiating precursor B-cells derived from spleens of 4-week-old Eμ-ret mice with TLR7 ligand, TLR8 ligand or TLR9 ligand showed reduced viable cell recovery, while increased cell expansion following TLR3 stimulation was observed. TLR9 stimulation induced long-term control of preleukemia and established leukemia in the same Eμ-ret model. Innate immune cells, in particular natural killer cells and macrophages were critical in mediating these effects and protected these mice in remission from relapse after leukemia re-challenge. Treatment with IFN-α- or IFN-γ-neutralizing antibodies reversed these effects, implying that proliferation or regression of leukemia initiating cells is interferon-dependent [55].

Loss of immunosurveillance within the bone marrow microenvironment is a further mechanism leading to immune evasion of ALL blasts. The “infective lymphoid recovery hypothesis” described by Richardson et al. focuses on the leukemia-promoting effects of delayed recurrent infections in childhood, causing heat-shock responses and transient involution of the thymus and other lymphoid organs, contributing to a decline in antitumor immuno-surveillance and the maturation arrest of B-cells secondary to glucocorticoid release in response to infection. The release of pro-inflammatory (Th1) cytokines in response to infection promotes cell survival and a hypermutable state, whereas the release of Th2 cytokines and interleukin-7 (IL-7) stimulates immature B-cell proliferation, including preleukemic cells [56].

Inflammation impacts preleukemic, stromal cells and the immune system in the bone marrow. An inflammation-microenvironment—leukemia initiating cell triad has been proposed as a key pathogenic mechanism in leukemia pathobiology [57]. The putative pediatric pB-ALL initiating niche shows gene expression signatures of bone marrow mesenchymal stem cell (MSC) with pro-inflammatory and suppressor niches [58]. The transcriptional profiles of MSC reveal two characteristic signatures: a CXCL12-low inflammatory and leukemia expansion-like niche that likely supports leukemic burden and a CXCL11 high immune-suppressive and leukemia-initiating cell (SLIC)-like niche, where LICs are likely sustained. The pro-inflammatory signature includes a large set of chemokines involved in neutrophil recruitment, metalloproteinase functional activation and leukocyte migration as well as pro-inflammatory molecules. The immune suppressive signature shows TLR signaling, cytokine mediated signaling and a negative regulation of leukocyte proliferation signatures with high expression of chemokines CXCL10 and CXCL11 as well as suppressor molecules like indole amine 2,3-dioxygenase (IDO1) and galectin 9 (LGALS9). In ALL, pro-inflammatory cytokine signaling including IL6, TNFa and IL-1b is involved in promoting leukemia initiation by cooperating with mesenchymal stromal cell (MSC) niches in selection and induction of *ETV6-RUNX1* pre-leukemic cells [59]. Key cells of the immune system that shape the leukemic microenvironment include immature myeloid populations, non-classical monocytes, macrophages, NK cells, and various subpopulations of T cells [60]. Immature myeloid populations involved in immunoregulation, referred to as myeloid-derived suppressor cells (MDSCs), include granulocytic (G) and monocytic (M) MSDCs [61, 62]. G-MDSCs inhibit the activity of T cells and natural killer (NK) cells through reactive oxygen species (ROS)-dependent mechanisms, and M-MDSCs through secretion of immunosuppressive cytokines (IL-10, TGF beta), arginase and nitric oxide. ALL blasts evade immunosurveillance escaping from NK cell recognition and lysis through downregulation of the ligands for NK cell-activating receptors (MICA/B, ULBP1, and NEC-2) [63], resist phagocytosis by macrophages by upregulating CD47 [64] and avoid a T cell response by triggering selected inhibitory checkpoints (TIM-3, CD200R) [65]. Increased Treg-cells have been linked with diminished potency of selected immunotherapies against pB-ALL, such as blinatumomab [66] or CAR-T cells [67]. The CXCL10/CXCL11/CXCR3 axis has been implicated in chemotherapy resistance and CNS infiltration in B-ALL [68]. Through single-cell transcriptome profiling Witkowski et al. elegantly compared the bone marrow immune microenvironment of healthy individuals and of B-ALL patients at diagnosis, remission, and relapse. The non-malignant immune bone marrow microenvironment in ALL patients is altered and shows enrichment with a non-classical monocyte subpopulation both at diagnosis and at relapse [69]. Differential gene expression analysis of leukemia-associated non-classical monocytes and their healthy counterpart show a leukemia-associated upregulation of genes involved in monocyte interactions with vascular endothelium during inflammation and vascular endothelial repair, such as *PECAM1*, *CD44*, *ITGA4*, *CX3CR1*, and *TNFSF10*, as well as significant downregulation of genes encoding subunits of HLA-DR. The differentiation of human classical monocytes toward the non-classical monocyte lineage is enhanced by the presence of human B-ALL. Acute leukemia interacts with the vascular endothelium leading to increased vascular permeability, and non-classical monocytes emerge in response to leukemia-induced inflammation to repair the damaged endothelium.

**Fig. 1:**
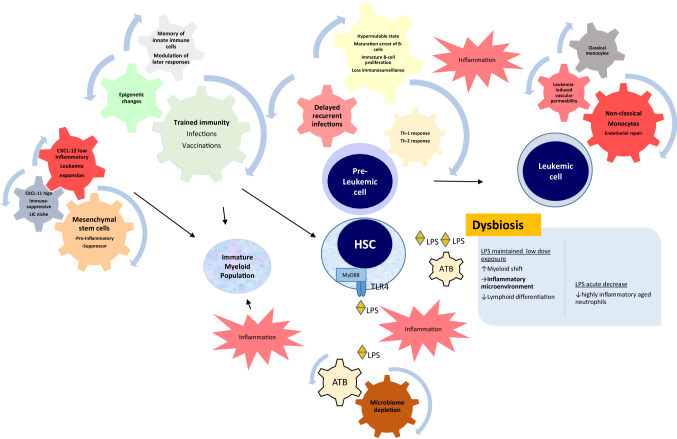
Innate immune cells undergo epigenetic modifications following infections or vaccination, which provide them with a memory, which subsequently modulates their response to a second infection exposure later in life (“trained immunity”). Delayed recurrent infections cause a decline in antitumor immuno-surveillance and the maturation arrest of B-cells secondary to glucocorticoid release in response to infection. The release of pro-inflammatory (Th1) cytokines promotes cell survival and a hypermutable state, whereas the release of Th2 cytokines and IL-7 stimulates immature B-cell proliferation, including preleukemic cells. The putative pediatric pB-ALL initiating niche shows gene expression signatures of bone marrow mesenchymal stem cell (MSC) with pro-inflammatory and suppressor niches. The differentiation of human classical monocytes towards the non-classical monocyte lineage is enhanced by the presence of human B-ALL. Acute leukemia interacts with the vascular endothelium leading to increased vascular permeability and non-classical monocytes emerge in response to leukemia-induced inflammation. Hematopoietic stem cells (HSC) express Toll-like receptors (TLRs), which are able to recognize microbial components and initiate innate immune responses. Bacterial lipopolysaccharide (LPS) binds the TLR4/Myd88 receptor complex on HSC and stimulates myeloid differentiation pathways, providing a means for rapid replenishment of the innate immune system during infection. The microbiome regulates neutrophil ageing and is required to balance hematopoiesis. A state of dysbiosis with persistent low plasmatic LPS leves can induce loss of lymphoid differentiation in HSC and a myeloid shift. One may hypothesize that whereas a healthy, species-rich microbiome balances normal hematopoiesis, the depletion of the microbiome by antibiotics followed by a state of dysbiosis, with persistently low plasmatic LPS levels may contribute to a lymphoid-myeloid shift and the generation of a myeloid inflammatory microenvironment, predisposing pre-leukemic cells to progression to overt pB-ALL

A further element gaining recognition in this puzzle is the role of the intestinal microbiome in pB-ALL [70, 71]. Within a human organism there are trillions of microbes that interact with the host constantly, mainly on the skin and on mucosal surfaces of the gastrointestinal tract. The gut microbiota, which mainly resides in the colon, develops during the first three years of life [72–74]. The structure of the gut, which contains a large mucosa surface consisting of a single epithelial cell layer made up of intestinal epithelial cells and intraepithelial lymphocytes, facilitates the interaction with the immune system. This gut-associated lymphoid tissue represents the largest component of the immune system in the body and influences immune responses locally and systemically. Bacterial fermentation of undigested dietary carbohydrates, mainly resistant starch and dietary fiber, produces short chain fatty acids (SCFA), primarily acetate, propionate, and butyrate, which are major players in the maintenance of gut physiology and integrity by promoting immune and metabolic homeostasis, with important anti-inflammatory effects [75], also influencing hematopoiesis through G protein–coupled receptors [76–78]. While alpha diversity is a measure of microbiome diversity applicable to a single sample, beta diversity is a measure of similarity or dissimilarity of two communities from different environments. Antibiotic-induced microbiota alterations have been shown to alter bile acid metabolism and insulin sensitivity in both humans and mice [79]. The specific composition of the microbiota alters the types and levels of SCFA that are formed, affecting numerous physiologic processes that are differentially modulated by acetate, propionate, and butyrate [80]. Besides the direct effect of antibiotics on the composition of the gut microbiota, antibiotics affect the manner in which this community interacts with the host and regulates basic physiological processes [81]. Bacterial byproducts can skew the dendritic cells of the gut-associated lymphoid tissue either towards a Treg-state, creating a local anti-inflammatory cytokine environment, or toward a Th17-state and creating an inflammatory phenotype [82]. The term dysbiosis refers to a persistent alteration of the gut microbiota, both in its composition and function [83], with a less diverse and less stable microbiota. Dysbiosis is commonly characterized by a reduced diversity of the phyla *Firmicutes* and *Bacteroidetes* and is often accompanied by an overgrowth of the family *Enterobacteriaceae*. Bacterial metabolites like the SCFA produced by *Bifidobacteriaceae* are involved in the host defense of colonic epithelium, affecting the mucus layer structure and consequently the host’s gastrointestinal barrier function [84]. As the microbiota composition changes, the altered microbial community will present a different repertoire of microbial-associated molecular patterns (MAMPS) to the receptors located in immune and epithelial cells, which results in an altered stimulation of receptors such as NOD1 and the Toll-like receptors (TLRs), which are involved in lymphoid tissue development, T cell differentiation, neutrophil priming and cytokine release [85]. HSC and progenitor cells express TLRs, which are able to recognize microbial components and initiate innate immune responses. Bacterial lipopolysaccharide (LPS) binds the TLR4/Myd88 receptor complex on hematopoietic stem cells and stimulates myeloid differentiation pathways, providing a means for rapid replenishment of the innate immune system during infection [86]. Nevertheless, repeated exposure to small amounts of LPS is harmful to long-term repopulating stem cells and induces the loss of the ability to differentiate towards lymphocytes with an enduring myeloid bias and lymphoid deficiency in HSC originating from marrow chronically exposed to LPS [87]. In Vicente-Dueñas et al.’s study [71] neither a single microbe nor the overall bacterial burden were connected with the development of pB-ALL, but in particular the depletion and subsequent reconstitution of the microbiome were significantly associated with pB-ALL. The intestinal microbiome of mice genetically predisposed to ALL through *Pax5* heterozygosity or *ETV6-RUNX1* fusion had a specific shape when compared to wild-type mice, and the alteration of this already distinct microbiome of *Pax5* heterozygous mice through antibiotic treatment was able to trigger pB-ALL in the absence of infectious stimuli [71]. Depleting the gut microbiome by antibiotic treatment did not prevent infection-driven pB-ALL development in *Pax5 1/2* mice in core facilities, but remarkably, this intervention promoted pB-ALL development in a specific pathogen-free environment, even in the absence of infectious stimuli [44]. Zhang et al. showed that microbiome regulates neutrophil ageing [88] and furthermore, that the microbiome is required to balance hematopoiesis. Microbiome depletion with antibiotics reduced the aged neutrophils, which are highly inflammatory. Neutrophil ageing is driven by the microbiome via TLR and myeloid differentiation factor88 (MyD88)-mediated signaling pathways, which transduce microbiome-derived signals. Microbiome depletion in specific-pathogen-free mice with prolonged broad-spectrum antibiotics resulted in significant and selective reductions of circulating aged neutrophils which were completely restored when TLR4 ligand LPS was added. In contrast, germ-free animals showed broad alterations in both innate and adaptive immune cells, with significantly reduced numbers of total and aged neutrophils. Treatment of germ-free mice with antibiotics did not further reduce the aged neutrophils but were partially restored when germ-free mice were reconstituted by fecal transplantation, suggesting that the microbiome is involved in a fine tuned and balanced regulation of immunity, influencing the proportion of highly active aged neutrophils and balancing hematopoiesis and the risk of tissue injury. These results are in line with Vicente-Dueñas et al.’s study [71], where the depletion and subsequent reconstitution of the microbiome by antibiotics in mice genetically predisposed to ALL through *Pax5* heterozygosity was significantly associated with pB-ALL in specific pathogen-free environment but not in core facilities. One may hypothesize that whereas a healthy, species-rich microbiome balances normal hematopoiesis, the depletion of the microbiome by antibiotics followed by a state of dysbiosis, with persistently low plasmatic LPS levels may contribute to a lymphoid-myeloid shift and the generation of a myeloid inflammatory microenvironment, predisposing pre-leukemic cells to progression to overt B-ALL (Fig. 1).

## Antibiotics in childhood, microbiome changes and data in childhood ALL patients

It has been shown in children, adults and in animal models that antibiotics dramatically alter the gut microbial composition [81, 89, 90]. Since antibiotics are among the most common medications prescribed to children in high-income countries [91], the potential for dysbiosis in the microbiome should be taken into account.

The table below (Table 1) summarizes standardized principles of antibiotic prescription for the most frequent infectious diseases in children obtaining care in an outpatient setting for the following diagnosis: acute rhinosinusitis, acute otitis media, bronchiolitis, pharyngitis, non-specific upper respiratory tract infections, and urinary tract infection.

**Table 1:** Most common infectious diseases in Pediatrics in the outpatient setting

Infectious disease	Management	Antibiotic treatment	References
Acute rhinosinusitis	Antibiotics are not guaranteed to help even if the causative agent is bacterial. If a bacterial infection is established: watchful waiting for up to 3 days may be offered	Only for children with acute bacterial sinusitis with severe or worsening disease. Amoxicillin or amoxicillin/clavulanate are first-line therapy	[92]
Acute otitis media	Mild cases with unilateral symptoms in children 6–23 months of age or unilateral or bilateral symptoms in children > 2 years may be appropriate for watchful waiting	Amoxicillin (first line therapy for children who have not received amoxicillin within the past 30 days). Amoxicillin/clavulanate if amoxicillin has been prescribed within the past 30 days, if concurrent purulent conjunctivitis is present, or if the child has a history of recurrent AOM unresponsive to amoxicillin	[93, 94]
Bronchiolitis	Most common lower respiratory tract infection in infants	Antibiotics are not helpful and should not be used	[95]
Pharyngitis	Streptococcal pharyngitis is primarily a disease of children 5–15 years old and is rare in children < 3 years. Rapid antigen detection test helpful	Amoxicillin or penicillin V first-line therapy Recommended treatment course for 10 days	[96]
Non-specific upper respiratory tract infection (URI)	At least 200 viruses known to URI Management should focus on symptomatic relief	Antibiotics should not be prescribed for these conditions	[97, 98]
Urinary tract infection (UTI)	Most common causative pathogen is *E. coli* (85% of cases). Initial antibiotic treatment should be based on local antimicrobial susceptibility patterns, then adjusted to antibiogram. Antibiotic treatment of asymptomatic bacteriuria in children is not recommended	Amoxicillin/clavulanate, trimethoprim-sulfamethoxazole, cefixime, cefpodoxime or cephalexin in children 2–24 months	[99, 100]

Yassour et al. performed an elegant longitudinal study on the infant gut microbiome and the impact of antibiotic treatment on bacterial strain diversity and stability. Based on whole-genome shotgun sequencing of monthly stool samples over 36 months in 39 children, half of whom received multiple courses of antibiotics during the first 3 years of life, it was shown that the microbiota of antibiotic-treated children had reduced bacterial and strain diversity and a less stable gut microbiome following antibiotic treatment. The most current evidence on gut microbiome dysbiosis in children after antibiotic treatment has been systematically reviewed by McDonnel et al. [101]. Only twelve studies met quality eligibility criteria for this systematic review and included five randomized controlled trials, five cohort studies and two cross-sectional studies that analyzed the relationship between antibiotics and gut microbiome dysbiosis, age 0–18 years, molecular techniques of assessment and outcomes of microbiome richness, diversity or composition. Five studies found a significant reduction in diversity and three of them a significant reduction in richness. Antibiotic exposure was associated with reduced microbiome diversity and richness, and with alterations in bacterial abundance, with reductions of *Bifidobacteria* and *Lactobacillus* and increases in Proteobacteria like *E. coli*. Macrolide exposure (azithromycin) was associated with reduced richness for twice as long as penicillin and a significant reduction in alpha-diversity. Quality of evidence in this rigorous meta-analysis was defined as good or fair [101], highlighting the need for further standardized research in this field [102].

The microbiome of pediatric ALL patients has a specific shape starting from the moment of diagnosis when compared to healthy controls. Several studies have shown that ALL patients differ in their gastrointestinal microflora at diagnosis when compared to healthy controls [103–108]. Additional evidence supporting this fact is that also the supragingival plaque is less rich and less diverse in pediatric ALL patients at diagnosis comparted with healthy controls [109]. As described by Oldenburg et al. at the onset of ALL, reduced diversity in the oral and gut microbiomes in ALL patients is already observed [110]. Further reduction of diversity occurs during treatment due to the administration of chemotherapeutics and antibiotics, with dominance of *Enteroccocaceae* being predictive of infections. Changes to the microbiome can be detected up to several years after completion of treatment, with possible implications for long-term health [110].

## Summary

The reviewed insights on the role of the innate immune responses in childhood pB-ALL models highlight the trained immunity concept as a possible target for future interventions towards prevention of pB-ALL in children. The role of the microbiome in normal hematopoiesis and the effect of dysbiosis on HSC is gaining increasing attention particularly since the observation that antibiotic treatment is able to trigger pB-ALL in the absence of infectious stimuli in *Pax5* heterozygous mice genetically predisposed to ALL [71]. Since antibiotics, which represent one of the most widely used medical interventions, alter the gut microbial composition and can cause a state of dysbiosis, this raises exciting epidemiological questions regarding the implications for antibiotic use in early life especially in infants with a a preleukemic “first hit”. Sheading light through a rigorous epidemiologic study on this piece of the puzzle may have broad implications for clinical practice.
